# Electrochemical determination of ciprofloxacin using a MIL-101/reduced graphene oxide-modified electrode

**DOI:** 10.3762/bjnano.17.35

**Published:** 2026-04-21

**Authors:** Nguyen Quang Man, Nguyen Ngoc Nghia, Nguyen Vinh Phu, Vo Thi Khanh Ly, Le Lam Son, Pham Khac Lieu, Le Thi Hong Phong, Nguyen Dinh Luyen, Dinh Quang Khieu

**Affiliations:** 1 University of Medicine and Pharmacy, Hue University, 530000, Vietnam; 2 University of Sciences, Hue University, 530000, Vietnam; 3 Department of Education and Training, Hue City, 530000, Vietnam; 4 Institute of Materials Sciences, Vietnam Academy of Science and Technology, 18 Hoang Quoc Viet, Cau Giay, Hanoi City,100000, Vietnam; 5 University of Education, Hue University, 530000, Vietnam

**Keywords:** ciprofloxacin, electrochemical analysis, MIL-101, reduced graphene oxide

## Abstract

An electrochemical sensor for the determination of ciprofloxacin (CPR) was developed using a glassy carbon electrode modified with MIL-101/reduced graphene oxide (MIL-101/rGO). MIL-101/rGO was synthesized via a facile ultrasonic-assisted method and characterized by various physicochemical techniques. The synergistic combination of MIL-101 with rGO significantly enhanced the electrocatalytic activity toward CPR oxidation. The electrochemical behavior of CPR on the MIL-101/rGO-modified electrode was systematically investigated using cyclic voltammetry and differential pulse voltammetry. Under optimized experimental conditions, the proposed sensor exhibited a linear response over 0.25–9.41 µM and a detection limit of 0.11 µM for CPR determination. The sensor also demonstrated good selectivity, satisfactory repeatability, and long-term stability. Furthermore, the method’s practical applicability was validated by the determination of CPR in pharmaceutical samples, yielding acceptable recoveries. These results indicate that the MIL-101/rGO-modified electrode provides a promising and efficient platform for the electrochemical sensing of CPR in pharmaceutical and environmental analysis.

## Introduction

Ciprofloxacin (C_17_H_18_FN_3_O_3_, CPR) is a fluoroquinolone antibiotic widely used in human and veterinary medicine to treat various bacterial infections [1]. Due to its high usage, incomplete metabolism, and environmental persistence, CPR residues are often found in pharmaceutical products, biological fluids, and aquatic environments [2–3]. The uncontrolled presence of ciprofloxacin not only poses risks to human health but also promotes the development of antibiotic-resistant bacteria, raising significant environmental and public health concerns [4]. Therefore, developing sensitive, selective, and affordable analytical methods for detecting ciprofloxacin is very important.

Conventional analytical techniques for CPR determination, including a fluorescence sensor based on CsPbBr_3_ quantum dots embedded in a molecularly imprinted polymer [5], and ultrasound-assisted magnetic dispersive micro-solid phase extraction based on carbon quantum dots/zeolite imidazolate framework-90/polyvinyl pyrrolidone/iron(II,III) oxide with high-performance liquid chromatography [6–7] or chromatography–tandem mass spectrometry [8] offer high accuracy and reliability. However, these techniques often need expensive equipment, complex sample preparation, skilled operators, and lengthy analysis times, which restrict their use for quick or in situ testing. In contrast, electrochemical sensors have become appealing alternatives because of their inherent benefits such as simplicity, low cost, rapid response, high sensitivity, and potential for miniaturization and field use.

The performance of electrochemical sensors heavily relies on the electrode’s surface properties. Recently, metal–organic frameworks (MOFs) have attracted significant interest in electrochemical sensing due to their highly ordered porous structures, very high surface areas, tunable pore sizes, and numerous active sites. Among these, MIL-101, a chromium-based MOF, is notable for its large pore volume, excellent chemical stability, and strong ability to adsorb organic molecules [9–11].

However, the poor electrical conductivity inherent to MIL-101 limits its direct use in electrochemical sensing. To address this limitation, hybridizing MOFs with conductive carbon materials has been extensively studied. Reduced graphene oxide (rGO) is especially appealing due to its high electrical conductivity [12], large specific surface area, mechanical stability [13], and strong π–π interactions with aromatic compounds like ciprofloxacin [14–15].

Integrating MIL-101 with rGO aims to combine the high surface area and adsorption capacity of MIL-101 with the excellent electrical conductivity and electron-transfer ability of rGO, creating a synergistic effect that boosts the electrochemical performance of the composite material. This combination can promote efficient analyte adsorption and fast charge transfer at the electrode interface. Consistent with this idea, Gu et al. reported that MIL-101/rGO composites exhibit enhanced electrocatalytic activity toward the reduction of metronidazole and the anodic stripping detection of Cd^2+^ and Pb^2+^, demonstrating a strong synergistic effect between MIL-101 and rGO in electrochemical sensing [16].

In this work, a MIL-101/rGO hybrid was successfully synthesized and used as a modifier for a glassy carbon electrode to develop a sensitive electrochemical sensor for CPR detection. The electrochemical behavior of CPR on the MIL-101/rGO-modified electrode was examined. The proposed sensor demonstrated enhanced electrocatalytic activity, high sensitivity, and a low detection limit for CPR. Additionally, the practical utility of the sensor was confirmed through its application in analyzing real samples, highlighting its potential for pharmaceutical and environmental monitoring.

## Experimental

### Materials

Ciprofloxacin (CPR) was obtained from a commercial supplier and used without additional purification. Chromium(III) nitrate nonahydrate (Cr(NO_3_)_3_·9H_2_O), terephthalic acid (H_2_BDC), graphite powder, potassium permanganate (KMnO_4_), sodium borohydride (NaBH_4_), sulfuric acid (H_2_SO_4_), hydrogen peroxide (H_2_O_2_), and other analytical-grade reagents were purchased from standard chemical suppliers. Britton–Robinson (BR) buffer solutions at different pH levels were prepared using a mixture of acetic acid, phosphoric acid, and boric acid, with pH adjusted using 0.2 M NaOH solution.

### Synthesis of reduced graphene oxide (rGO), MIL-101, and MIL-101/rGO

Graphene oxide (GO) was synthesized from natural graphite using a modified Hummers’ method, as described in [17]. MIL-101(Cr) was prepared through a hydrothermal reaction by dissolving terephthalic acid (H_2_BDC, 1.66 g) and chromium(III) nitrate nonahydrate (Cr(NO_3_)_3_·9H_2_O, 4.0 g) in 48 mL of deionized water, followed by the addition of 2 mL of hydrofluoric acid. The mixture was transferred to a Teflon-lined stainless-steel autoclave and heated at 200 °C for 8 h [18]. After cooling to room temperature, a highly crystalline green chromium terephthalate powder (MIL-101(Cr)) was collected by filtration, thoroughly washed with deionized water, and dried before use.

The MIL-101/rGO composites were synthesized following a reported procedure with slight modifications [18]. Briefly, dried MIL-101(Cr) (0.1 g) was dispersed in 20 mL of deionized water and ultrasonicated for 30 min to obtain a homogeneous suspension. Subsequently, a predetermined amount of GO was added to the MIL-101 suspension under continuous stirring, followed by ultrasonication for 30 min. Sodium borohydride (NaBH_4_) was then introduced into the mixture, which was heated at 60 °C and stirred continuously for 12 h to reduce GO and form the MIL-101/rGO composite (Table 1).

**Table 1:** The composition for MIL-101/rGO.

Notation	MIL-101 (mg)	GO (mg)	NaBH_4_ (mg)

MIL-101/rGO(10)	100	10	35
MIL-101/rGO(5)	100	20	70
MIL-101/rGO(3.3)	100	30	105
MIL-101/rGO(2.5)	100	40	140
MIL-101/rGO(2)	100	50	175

After the reaction was completed, the product was collected by filtration, washed several times with deionized water to remove residual reagents, and dried at 60 °C for 24 h before use. The resulting composites were labeled as MIL-101/rGO(10), MIL-101/rGO(5), MIL-101/rGO(3.3), MIL-101/rGO(2.5), and MIL-101/rGO(2), where the numbers in parentheses indicate the mass ratio of MIL-101 to GO. For comparison, reduced graphene oxide (rGO) was also prepared using the same reduction process without MIL-101.

### Equipment

The X-ray diffraction analyses were conducted using a D8 Advance Bruker (Germany). Morphology and elemental mapping were measured with a Hitachi S-4800 FESEM (Japan) equipped with an energy-dispersive X-ray (EDX) system. Raman spectroscopy was performed on an Xplora Plus instrument (Horiba, Japan) with a stimulating light wavelength of 785 nm. Electrochemical impedance spectra (EIS) were recorded using an Autolab PGSTAT302N system. Electrochemical experiments were carried out on a CPA-HH5 workstation (Vietnam). A platinum wire served as the counter electrode, and a glassy carbon electrode was used as the working electrode.

### Preparation of the modified electrode

Before modification, the glassy carbon electrode (GCE) was polished successively with alumina slurry (0.05 µm) on a polishing cloth, thoroughly rinsed with deionized water, and sonicated in ethanol and water to remove residual impurities. To prepare the modified electrode, a specific amount of MIL-101/rGO composite was dispersed in deionized water using ultrasonication to create a uniform suspension (1 mg·mL^−1^). An aliquot of this suspension (5 µL) was drop-cast onto the cleaned GCE surface and allowed to dry naturally at room temperature to form the MIL-101/rGO-modified electrode. The modified electrode was then gently rinsed with deionized water before conducting electrochemical measurements.

### Electrochemical measurements

Electrochemical measurements were conducted using a standard three-electrode setup connected to an electrochemical workstation. Cyclic voltammetry (CV) and differential pulse voltammetry (DPV) were employed to analyze the electrochemical behavior of CPR. All tests took place in BR buffer solution at room temperature under optimized pH conditions. DPV parameters, including accumulation potential (*E*_acc_), accumulation time (*t*_acc_), pulse amplitude (Δ*E*), and voltage step (*U*_step_), were optimized before conducting the analytical measurements.

### Real sample preparation

Pharmaceutical samples containing CPR were prepared by carefully weighing and finely grinding the tablets (or powder for oral suspension). A suitable amount of the powdered sample was dissolved in deionized water and sonicated to ensure complete dissolution. The resulting solution was filtered to remove insoluble excipients and properly diluted with BR buffer (pH 4) before electrochemical analysis. The CPR content in the pharmaceutical samples was measured using the standard addition method.

### Data analysis

All electrochemical measurements were performed in triplicate. Calibration curves were constructed from DPV peak currents versus CPR concentrations. The limit of detection (LOD) was calculated based on the formula 3*S*/*b* where *S* is the standard deviation of the blank signal (or the standard deviation of the intercept of the calibration curve), and *b* is the slope of the calibration curve [19]. Repeatability, reproducibility, selectivity, and stability of the sensor were evaluated using relative standard deviation (RSD) values.

## Results and Discussion

### Materials characterization

**Figure 1:**
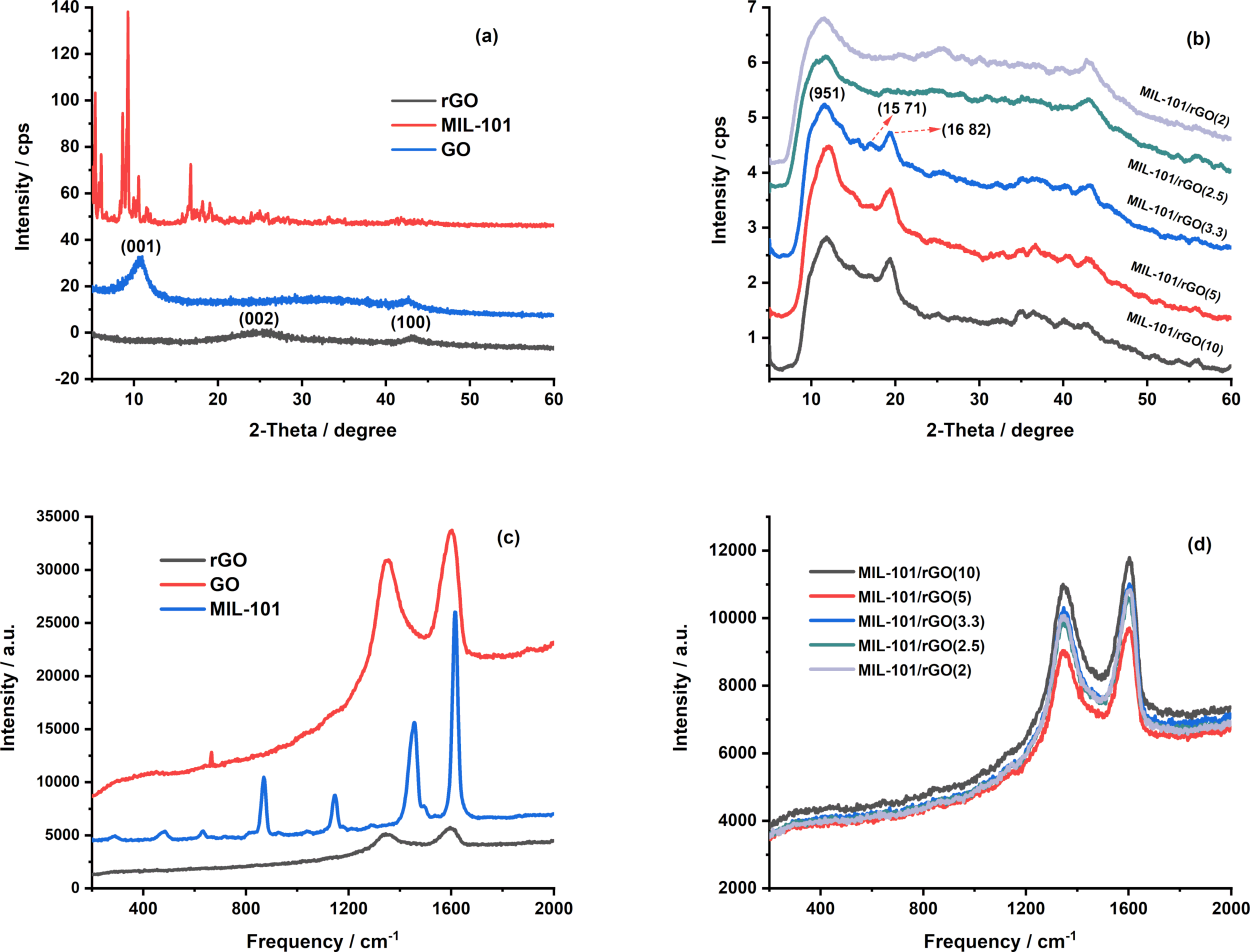
(a) X-ray diffraction (XRD) patterns of graphene oxide (GO), reduced graphene oxide (rGO), and MIL-101, (b) X-ray patterns of MIL-101/rGO with various mass ratios, (c) Raman spectra of GO, rGO and MIL-101, and (d) Raman spectra of MIL-101/rGO with various mass ratios.

Figure 1a presents the XRD patterns of GO, rGO, and pristine MIL-101, while Figure 1b shows the diffraction patterns of MIL-101/rGO composites prepared with different MIL-101/rGO mass ratios (as indicated in parentheses). In Figure 1a, GO exhibits characteristic diffraction peaks at 2θ values of 10.9° and 42.7°, corresponding to the (001) plane, which arise from the increased interlayer spacing caused by abundant oxygen-containing functional groups [20]. After reduction, this peak disappears, and broad reflections centered at 2θ values of 25.8° and 43.1° appear for rGO, attributed to the (002) and (100) planes of graphitic carbon, confirming the successful reduction of GO and partial restoration of the sp^2^ carbon network [21].

The XRD pattern of the synthesized MIL-101 shows several characteristic diffraction peaks located at 2θ ≈ 5.2°, 5.6°, 5.9°, 8.1°, 8.4°, 9.1°, 10.3°, 16.5°, and 18.0°, which can be indexed to the (333), (440), (442), (733), (660), (911), (951), (15 7 1), and (16 8 2) crystal planes of MIL-101(Cr) according CCCD No. 605510 (Supporting Information File 1, Figure S1) confirming the successful formation of the crystalline MOF framework. As shown in Figure 1b, all MIL-101/rGO composites retain the characteristic angle diffraction peaks of MIL-101 at 2θ = 11.7°, 15.6°, 17.0°, and 19.3°, consistent with [22], indicating that the crystalline framework of MIL-101 remains intact after mixing with rGO, regardless of the mass ratio. Meanwhile, the broad diffraction feature associated with rGO becomes more prominent with increasing rGO content, reflecting the growing contribution of the disordered graphitic phase. The XRD results from both figures confirm the successful formation of MIL-101/rGO composites. This structural combination is beneficial for electrochemical applications because rGO improves electrical conductivity and promotes charge transfer.

The Raman spectra of GO, rGO, MIL-101, and the MIL-101/rGO composites are presented in Figure 1a,c. As shown in Figure 1c, GO exhibits two characteristic bands at ≈1345 cm^−1^ (D band) and ≈1599 cm^−1^ (G band), which are associated with structural defects/disorder and the in-plane vibration of sp^2^-bonded carbon atoms, respectively [23]. After chemical reduction, rGO shows a slight shift of these bands to ≈1340 cm^−1^ (D) and ≈1592 cm^−1^ (G), accompanied by an increase in the intensity ratio *I*_D_/*I*_G_ from 0.91 (GO) to 0.99 (rGO). This increase indicates the partial restoration of sp^2^ carbon domains together with the generation of new, smaller graphitic domains, which is typical for reduced graphene oxide. In contrast, pristine MIL-101 displays multiple Raman bands in the low- and mid-frequency regions (280, 473, 629, 809, 866, 925, 1037, 1145, 1283, 1454, and 1611 cm^−1^), which can be assigned to lattice vibrations of the metal–oxygen clusters and organic linker modes, confirming the successful formation of the MIL-101 framework [24]. For the MIL-101/rGO composites (Figure 1d), the Raman spectra are dominated by the D and G bands of rGO located at ≈1340 and ≈1600 cm^−1^, while the characteristic bands of MIL-101 become much weaker or partially overlapped due to the strong Raman response of the carbon phase. The *I*_D_/*I*_G_ ratios of the composites range from 0.89 to 0.94, depending on the rGO content. Notably, these values are slightly lower than that of pure rGO, suggesting that the incorporation of MIL-101 can partially promote better ordering of sp^2^ domains through interfacial interactions between MIL-101 and rGO sheets.

**Figure 2:**
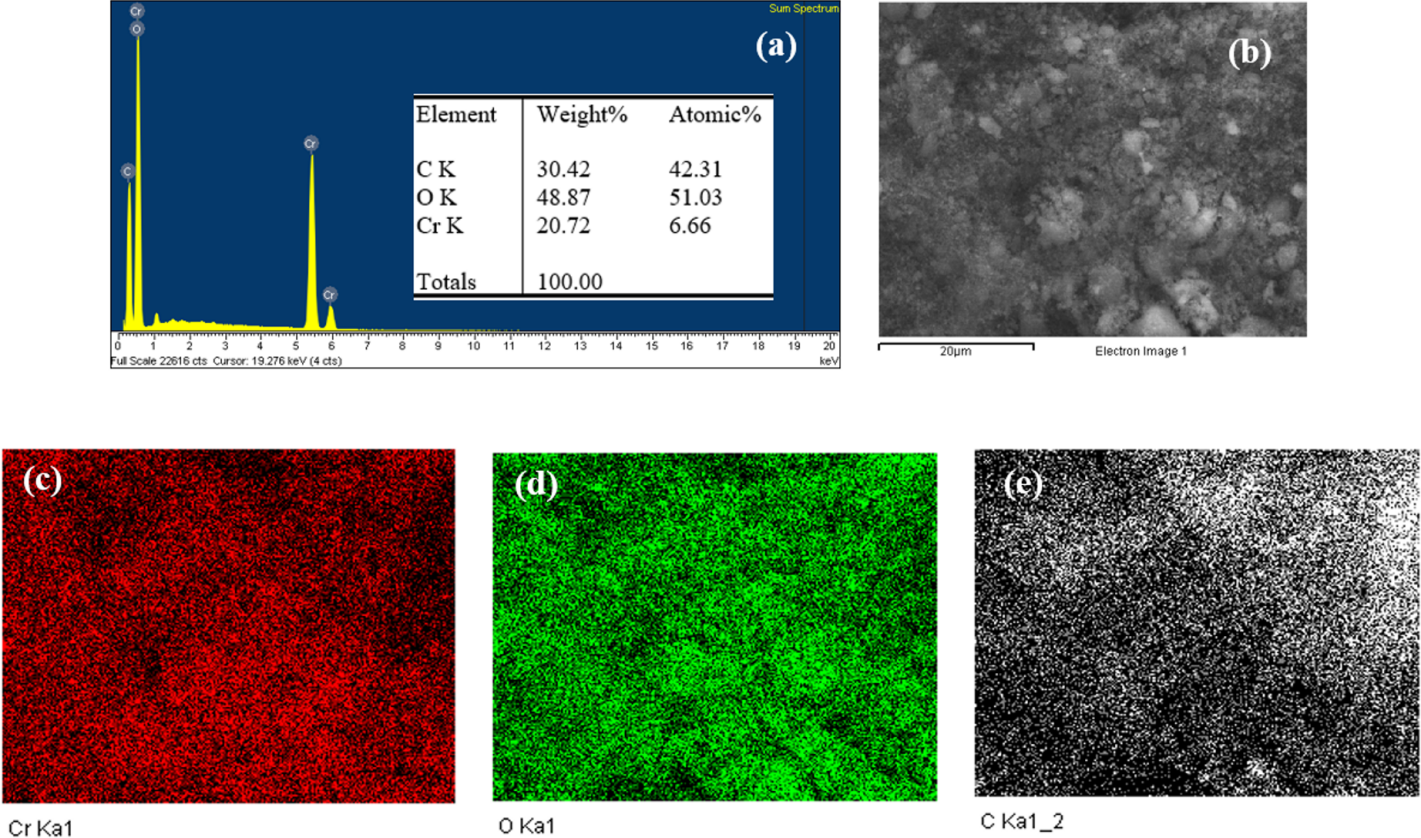
EDX elemental mapping and elemental composition of MIL-101/rGO (3.3), (a) EDX spectrum, (b) electron image, (c) chromium element mapping, (d) oxygen element mapping, and (e) carbon element mapping.

The EDX results confirm the successful formation of the MIL-101/rGO composite through the presence of C, O, and Cr as the main constituent elements. As summarized in the elemental composition table, carbon (C) accounts for 30.42 wt % (42.31 atom %), which mainly originates from the rGO sheets and the organic linker of MIL-101, indicating the effective incorporation of rGO into the composite matrix. Oxygen (O) is the most abundant element with 48.87 wt % (51.03 atom %), consistent with the presence of metal–oxygen clusters in MIL-101 as well as residual oxygen-containing functional groups on rGO. Chromium (Cr), the metal center of MIL-101, is detected at 20.72 wt % (6.66 atom %), confirming the successful introduction of the MIL-101 framework (Figure 2).

**Figure 3:**
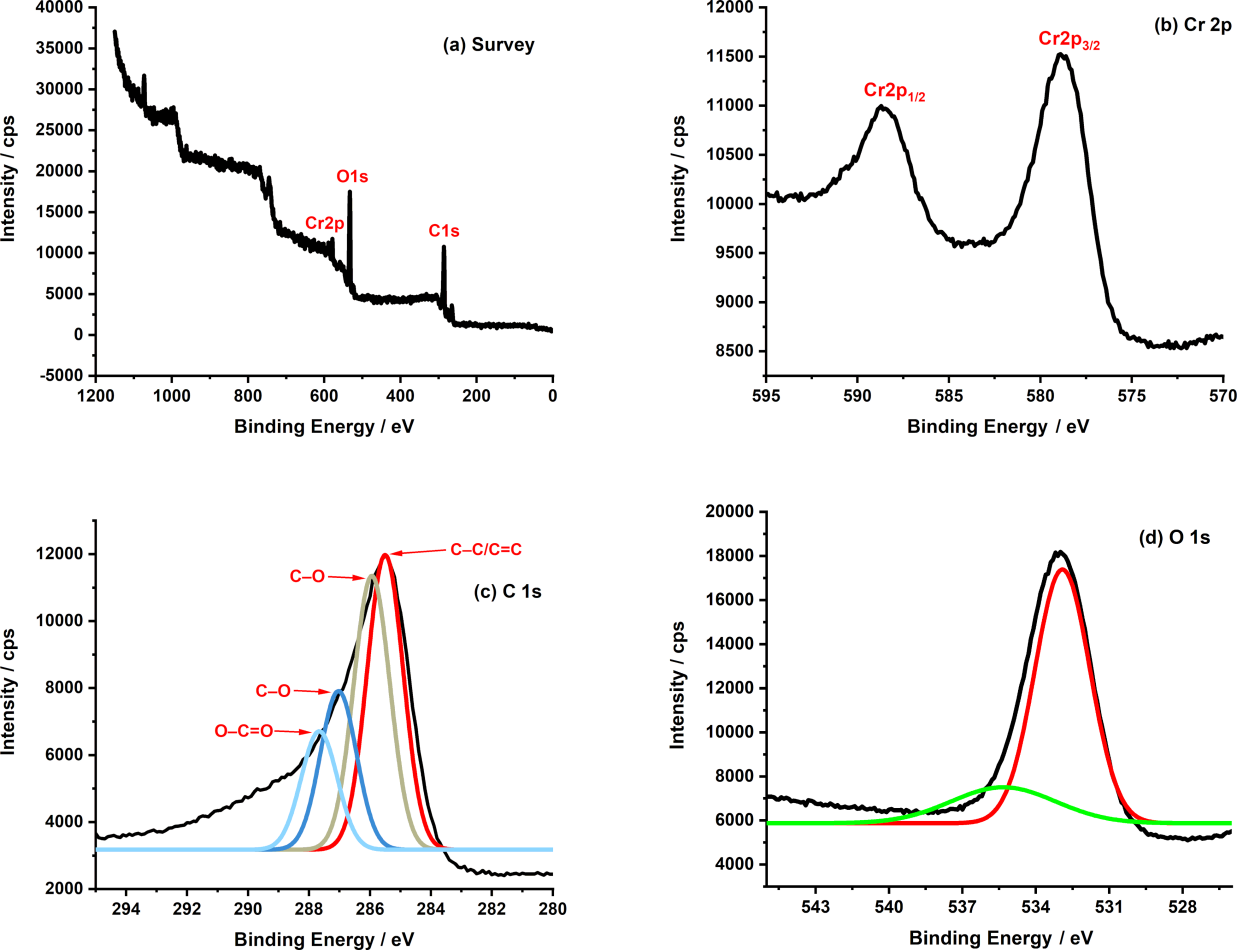
XPS spectra of the MIL-101/rGO (3.3): (a) survey spectrum, (b) high-resolution Cr 2p spectrum, (c) high-resolution C 1s spectrum, and (d) high-resolution O 1s spectrum.

The chemical composition and electronic states of the MIL-101/rGO composite were investigated by X-ray photoelectron spectroscopy (XPS). The survey spectrum (Figure 3a) confirms the presence of Cr, C, and O, which is consistent with the expected composition of MIL-101(Cr) integrated with reduced graphene oxide (rGO). No obvious extraneous elemental signals were observed in the survey spectrum, suggesting that the synthesized composite is predominantly composed of the expected elements within the detection limit of XPS. The high-resolution Cr 2p spectrum (Figure 3b) shows two prominent peaks at approximately 577.9 and 589.0 eV, representing the Cr 2p3/2 and Cr 2p1/2 spin–orbit components, respectively. The energy gap between these peaks (≈11.1 eV) is typical of Cr^3+^ species coordinated with oxygen in the Cr–O clusters of the MIL-101 framework [25]. Importantly, no signals related to Cr^6+^ species (usually observed at higher binding energies) are detected, confirming that chromium remains mainly in the trivalent oxidation state. This indicates that the Cr environment in MIL-101 stays intact after integration with rGO. The C 1s spectrum (Figure 3c) can be decomposed into several components representing different carbon bonding environments. The primary peak at about 285.6 eV corresponds to C–C/C=C bonds found in the sp^2^-hybridized carbon of the graphene lattice, indicating the presence of rGO sheets. Other components at around 285.9–287.1 eV are assigned to C–O functional groups, such as hydroxy or epoxy groups, while the peak near 287.7 eV can be attributed to O–C=O groups, originating from the carboxylate ligands of the terephthalate linkers in MIL-101 and residual oxygen functionalities on rGO [26]. The presence of these oxygen groups suggests that the reduction of graphene oxide is partial and that surface functional groups remain, helping to facilitate interactions between rGO sheets and MIL-101 particles. The O 1s spectrum (Figure 3d) shows two main contributions. The peak around 532.9 eV is due to Cr–O bonds within the metal–oxo clusters of MIL-101(Cr), confirming the formation of the MOF framework. The second feature at approximately 535.8 eV can be attributed to oxygen species in C–O groups, hydroxy groups, or adsorbed water molecules associated with the rGO surface and the porous MOF structure [27]. The presence of these oxygen-containing species further supports the coexistence of MIL-101 and rGO within the composite. Overall, the XPS results confirm the successful integration of MIL-101(Cr) and rGO, while preserving the Cr^3+^ oxidation state and the characteristic coordination environment of the MIL-101 framework.

**Figure 4:**
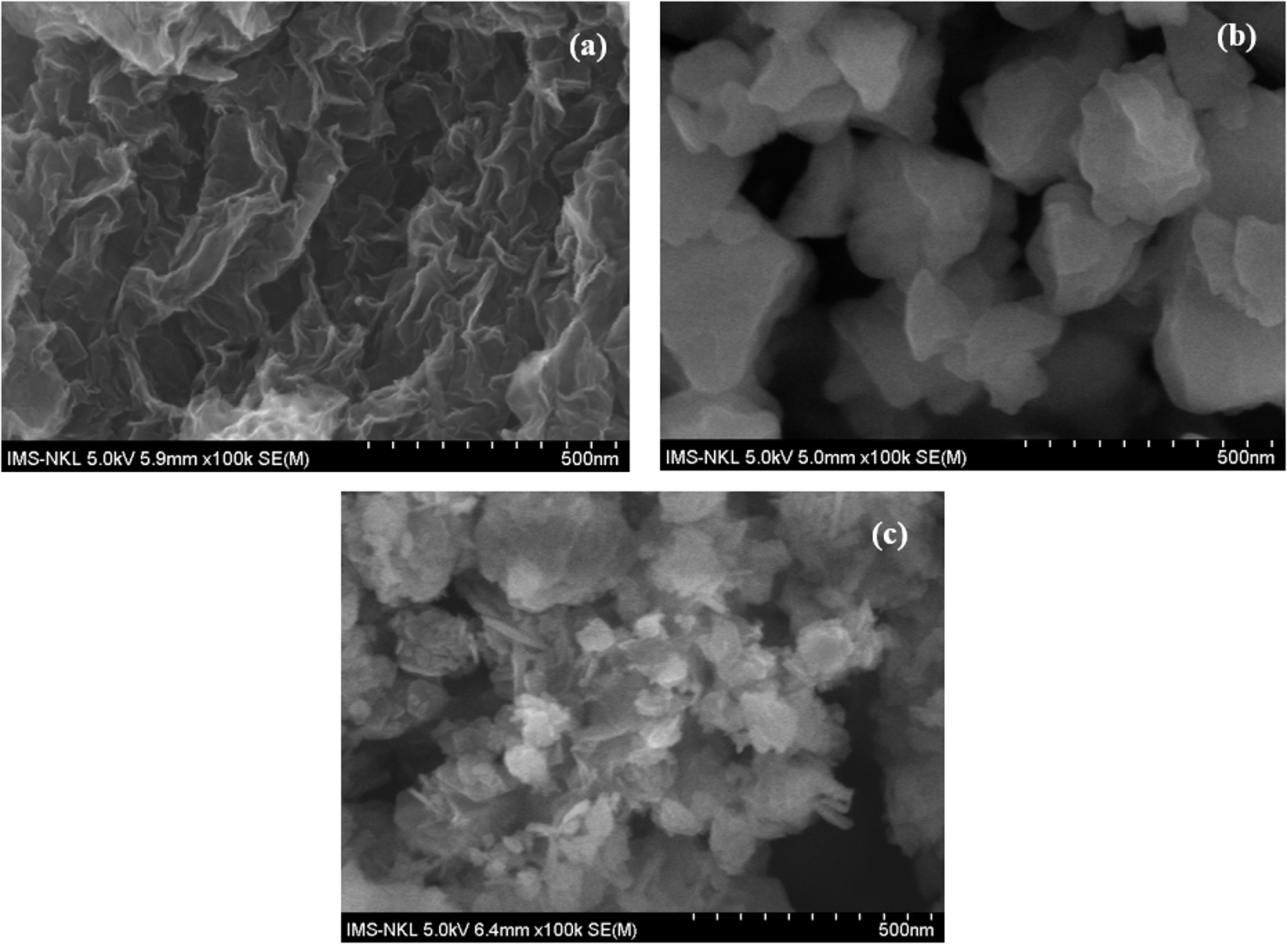
SEM observation of (a) rGO, (b) MIL-101, and (c) MIL-101(Cr)/rGO (3.3).

The SEM images of rGO, MIL-101, and the MIL-101/rGO composite are shown in Figure 4. The rGO sample (Figure 4a) displays a typical wrinkled, sheet-like morphology that offers a large surface area and a conductive substrate for material deposition. The pristine MIL-101 (Figure 4b) consists of irregular polyhedral particles that tend to aggregate, forming relatively compact clusters. In contrast, the MIL-101/rGO composite (Figure 4c) reveals that MIL-101 particles are dispersed on the wrinkled rGO sheets. The rGO sheets serve as a supporting scaffold, helping to separate the MIL-101 particles and reduce their aggregation to some extent. This structural characteristic benefits the dispersion of the MOF particles and enhances electron transfer within the composite material.

### Electrochemical determination of ciprofloxacin (CPR) at MIL-101/rGO/GCE

#### Electrochemical behavior of CPR

**Figure 5:**
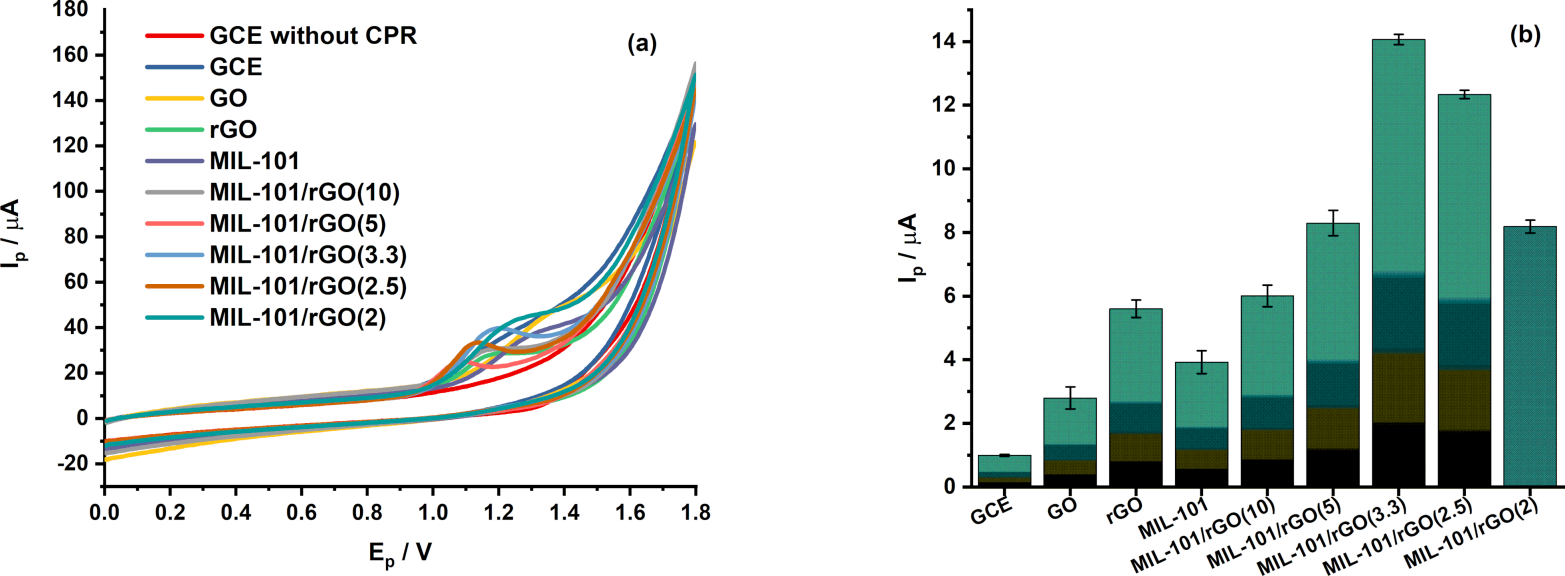
(a) Cyclic voltammograms (CVs) recorded at different electrodes in 0.1 M BRS buffer (pH 4) containing 15 µM CPR at a scan rate of 0.1 V/s and (b) comparison of the corresponding peak currents obtained at various electrodes (*n* = 3).

Figure 5a compares the cyclic voltammograms of CPR recorded at different electrodes in 0.1 M BR buffer (pH 4) (see Supporting Information File 1, Figure S2). A weak and poorly defined oxidation peak is observed at the bare GCE, indicating sluggish electron-transfer kinetics. Upon modification with MIL-101 or rGO, the anodic peak current increases noticeably, reflecting improved adsorption and conductivity. The obtained peak potentials (*E*_p_) for GCE, GO/GCE, rGO/GCE, MIL-101/GCE, MIL-101/rGO(10)/GCE, MIL-101/rGO(5)/GCE, MIL-101/rGO(3.3)/GCE, MIL-101/rGO(2.5)/GCE, and MIL-101/rGO(2)/GCE were 1.203, 1.150, 1.166, 1.288, 1.153, 1.072, 1.175, 1.125, and 1.219 V, respectively. In general, after modification of the electrode surface with these materials, the oxidation peak potential of CPR shifted to lower potentials compared with bare GCE (1.203 V), accompanied by a significant increase in peak current. This behavior indicates that the modified materials can effectively enhance the electrocatalytic activity toward CPR oxidation. Notably, the MIL-101/rGO composite electrodes exhibit a much higher oxidation response than either MIL-101/GCE or rGO/GCE alone, demonstrating an apparent synergistic effect between the high surface area and adsorption capability of MIL-101 and the excellent electrical conductivity of rGO. Among the investigated compositions, MIL-101/rGO(3.3)/GCE provides the highest and most reproducible peak current (Figure 5b), and was therefore selected for subsequent experiments.

#### Effect of pH

**Figure 6:**
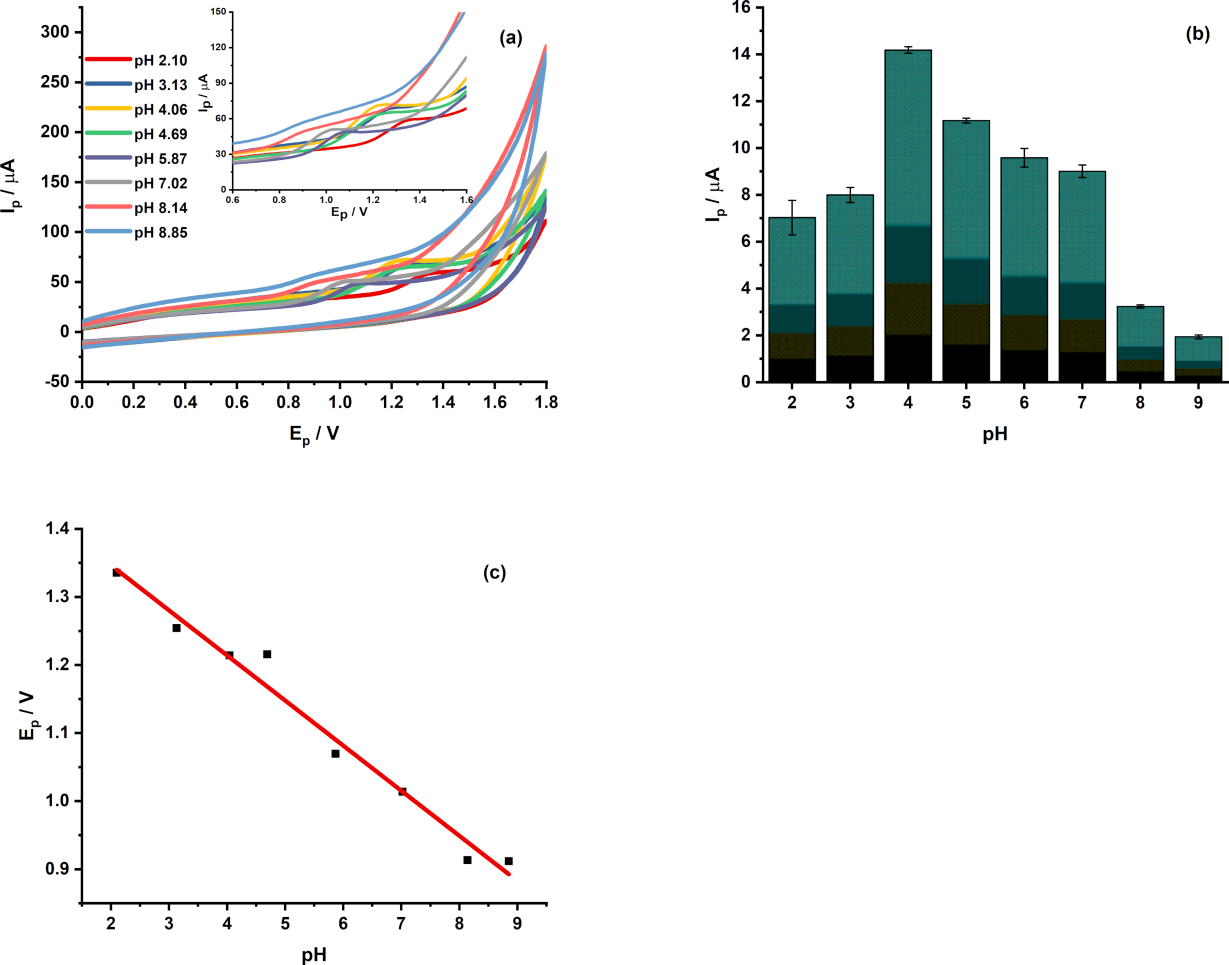
(a) CVs of MIL-101/rGO(3.3)/GCE recorded in 0.1 M BR buffer over the pH range of 2–9 containing 15 µM CPR at a scan rate of 0.1 V·s^−1^ and the inset shows the magnified view of the peak potential region, (b) variation of the oxidation peak current with pH, and (c) dependence of the oxidation peak potential on pH.

The impact of solution pH on the electrochemical oxidation of CPR was studied over a pH range of 2–9 (Figure 6). The oxidation peak current rises with pH up to 4, then gradually declines at higher pH levels, showing that both CPR’s protonation state and its interaction with the electrode significantly influence the electrochemical response (Supporting Information File 1, Figure S3). As a result, pH 4 was selected as the optimal condition. Additionally, the oxidation peak potential shifts linearly toward less positive values as pH increases. The linear relationship between peak potential and pH indicates that protons participate in the electrode process.




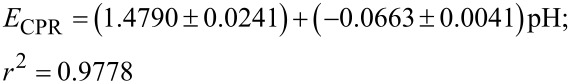




The slope of approximately 0.066 V·pH^−1^ is close to the theoretical Nernstian value, indicating that the number of transferred electrons is similar to that of protons. This result suggests a proton-coupled electron-transfer mechanism for CPR oxidation.

#### Effect of scan rate and kinetic analysis

**Figure 7:**
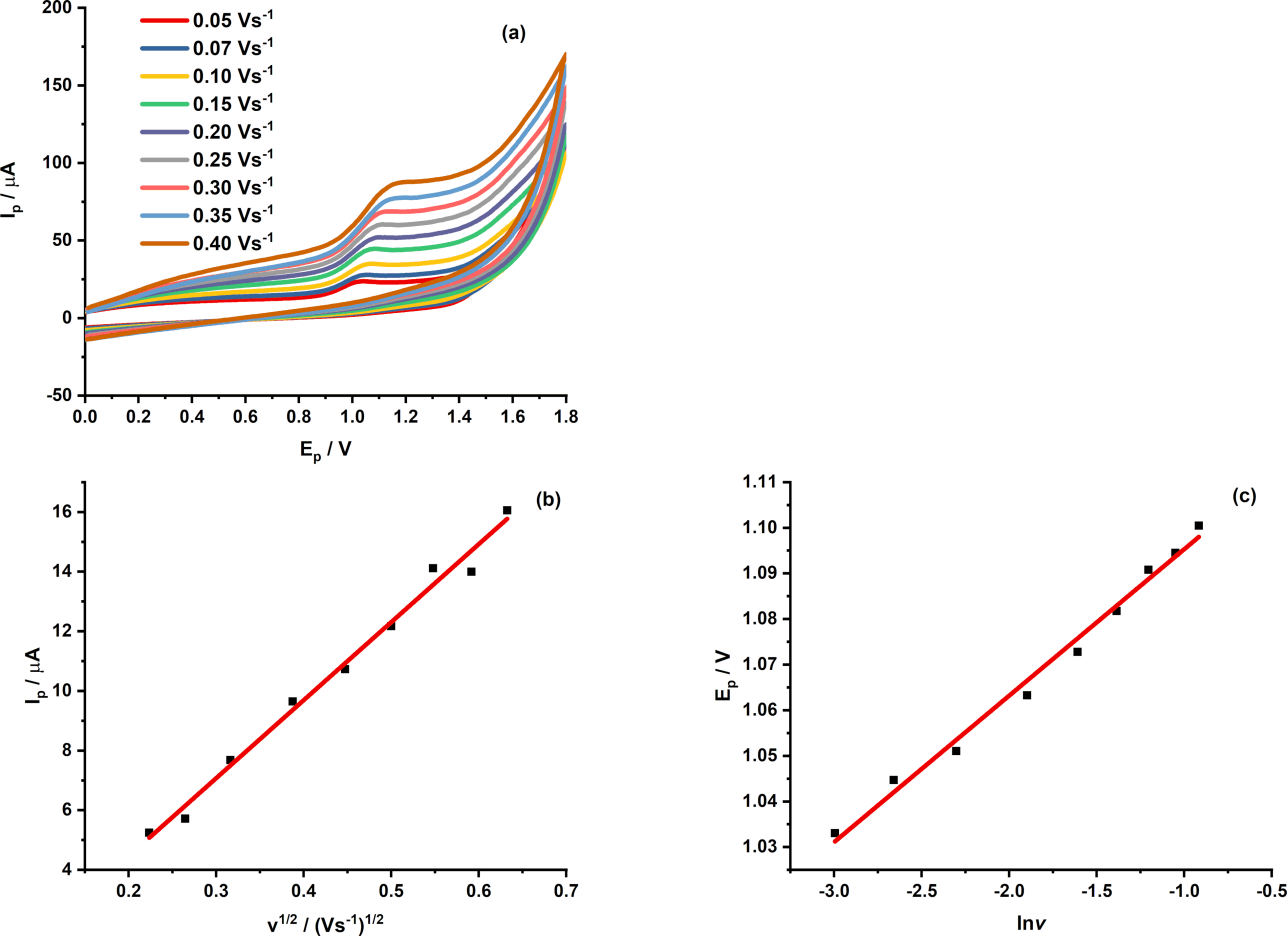
(a) Cyclic voltammograms of MIL-101/rGO(3.3)/GCE recorded in 0.1 M BR buffer (pH 4) containing 15 µM ciprofloxacin at scan rates from 0.05 to 0.40 V·s^−1^; (b) linear relationship between the peak current and the square root of scan rate (v^1/2^), and (c) linear dependence of the peak potential of ciprofloxacin on the natural logarithm of scan rate (ln *v*).

The effect of scan rate on the electrochemical response of CPR at MIL-101/rGO(3.3)/GCE was examined over the range of 0.05–0.40 V·s^−1^ (Figure 7). The oxidation peak current increases linearly with the square root of the scan rate (Figure 7a), indicating that CPR oxidation is mainly controlled by diffusion [28].




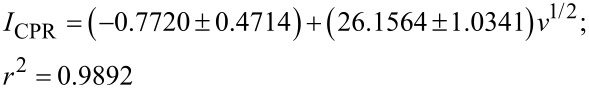




Furthermore, based on Laviron’s theory [29], the linear dependence of peak potential on the natural logarithm of scan rate confirms the irreversible nature of the oxidation process (Figure 7c).




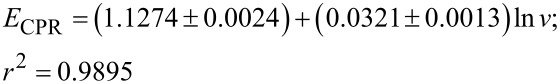




For an irreversible electrochemical system, the charge-transfer coefficient (α) was assumed to be 0.5, as is common in the literature [30].

**Scheme 1:**
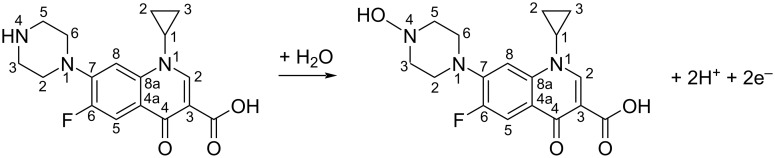
The oxidation mechanism of CPR at MIL-101/rGO/CGE.

From the slope of the *E*_CPR_–ln *v* plot, the product of α and the number of transferred electrons (*n*) was calculated to be 0.7999, leading to an estimated *n* value of approximately 1.6. Although this does not correspond to an integer, it reasonably indicates the involvement of two electrons in the oxidation process. When combined with the pH-dependent study, the results further suggest that the electrochemical oxidation of CPR at the modified electrode likely involves a two-electron, two-proton transfer process, aligning with previously reported electrochemical oxidation mechanisms of CPR [31]. The proposed oxidation pathway of CPR is schematically illustrated in Scheme 1.

#### Optimization of DPV parameters

To achieve maximum sensitivity, the parameters for differential pulse voltammetry (DPV) were systematically optimized. The accumulation potential, accumulation time, pulse amplitude, and potential step were investigated individually (Supporting Information File 1, Figure S4–S7). The optimal conditions were determined to be an accumulation potential of 0 V, an accumulation time of 4 s, a pulse amplitude of 0.11 V, and a voltage step of 0.010 V. Under these conditions, the oxidation peak current of CPR reached its maximum while maintaining a good peak shape and stable signal.

#### Analytical performance and linear range

**Figure 8:**
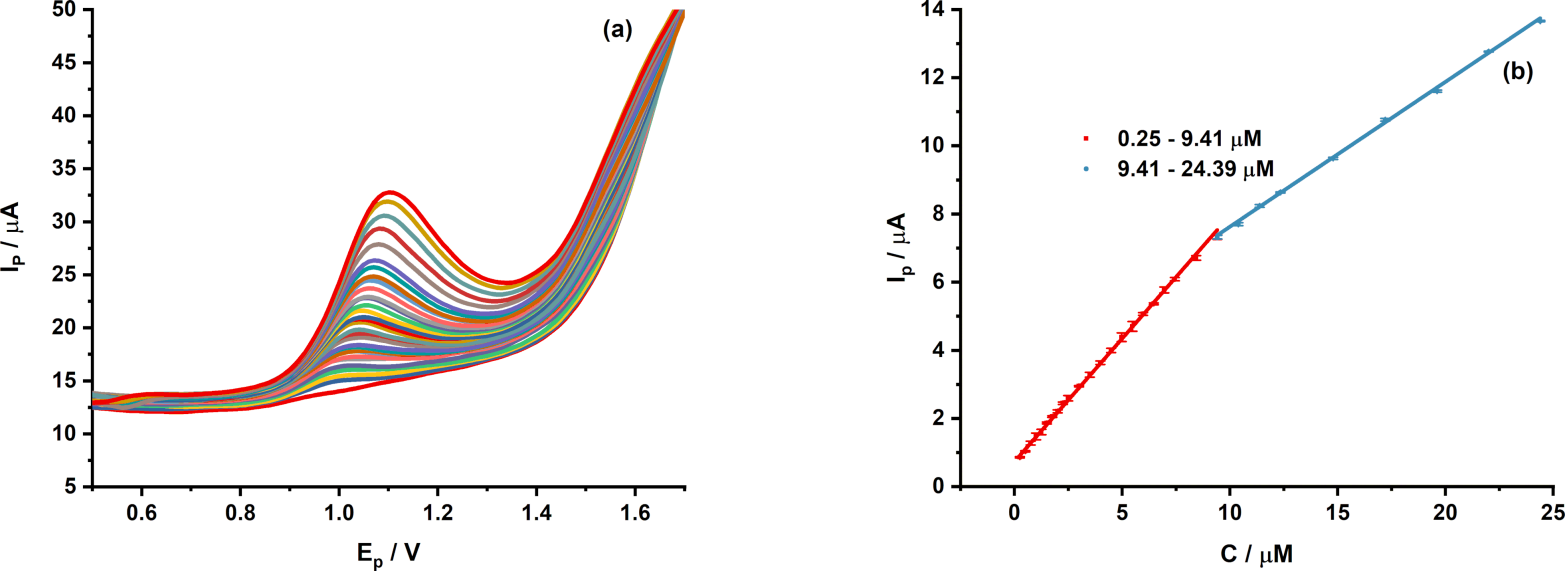
(a) DPV curves of MIL-101/rGO(3.3)/GCE recorded in 0.1 M BRS buffer (pH 4) at CPR concentrations ranging from 0.25 to 24.39 µM and (b) corresponding calibration plots of peak current versus ciprofloxacin concentration.

The analytical performance of the proposed sensor was evaluated by recording the DPV responses at increasing analyte concentrations (Figure 8a). As the CPR concentration rose, the peak current steadily increased, indicating that the electrochemical signal highly depends on the CPR concentration. The calibration plot of peak current versus concentration is shown in Figure 8b. Two linear regions are observed within the concentration ranges of 0.25–9.41 and 9.41–24.39 µM.

The corresponding regression equations are described as follows




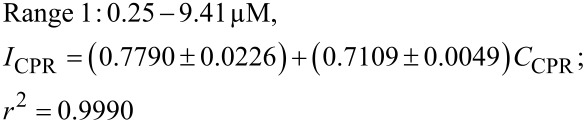







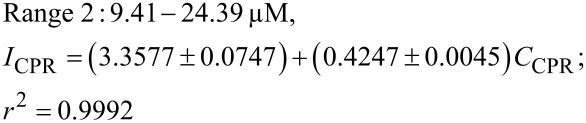




The presence of two linear ranges indicates a change in the interaction between the CPR molecules and the electrode surface at higher concentrations. At lower concentrations, CPR molecules are effectively adsorbed onto the active sites of the MIL-101/rGO-modified electrode, resulting in higher sensitivity. However, as the concentration increases, the number of available active sites gradually decreases due to partial surface occupation by CPR molecules. As a result, the slope of the calibration curve decreases slightly in the higher-concentration range. This behavior shows the beginning saturation of active sites on the electrode surface, which is common in adsorption-controlled electrochemical sensing systems. However, a strong linear relationship is still maintained across a wide concentration range, demonstrating the good analytical performance of the proposed sensor for quantitative analysis.

The limit of detection (LOD_CPR_) and the limit of quantification (LOQ_CPR_) were 0.11 and 0.36 µM, respectively. Table 2 shows the LOD and linear range of the proposed DPV and compares them with published reports. The LOD of the proposed sensor was comparable to previous sensors. The advantage of using the MIL-101(Cr)/rGO composite comes from the synergistic combination of its two components. MIL-101(Cr) has a high surface area and abundant porous structure, providing numerous active sites and strong adsorption capability for the target molecules; rGO offers high electrical conductivity, facilitating rapid electron transfer.

**Table 2:** Comparison of the analytical performance of the proposed MIL-101/rGO/GCE with previously reported electrochemical sensors for CPR determination.

Electrodes	Methods	Linear range (µM)	LOD (µM)	Real samples	Ref.

CB–PLA (3D-printed) sensor	–	1.0–12.5	0.3	tap water and synthetic urine	[32]
Poly [Mn(Chr)_3_]Cl_2_/PGE	SWV	1–200	0.536	pharmaceutical and urine samples	[33]
AgNPs-CB-Ch/GCE	SWV	3.1–24.8; 36.9–130.3	0.48	synthetic urine samples	[34]
GaTCPP(Ni)-Nafion/SPCE	DPV	200–1000	118	pharmaceutical samples	[35]
MWCNT/GCE	chronoamperometric	40–1000	6	urine and serum samples	[36]
CNSs/GCE	DPV	0.5–5.0	0.15	water sample and medicinal formulations	[37]
{[Co(HL)(bix)]·H_2_O}*_n_*/GCE	DPV	2–20	0.135	water samples	[38]
[Co(HL)(bimb)·H_2_O]}*_n_*/GCE	DPV	1–14	0.082	water samples	[38]
MIL-101/rGO/GCE	DPV	0.25–9.41	0.110	pharmaceutical formulations	this study

#### Repeatability, reproducibility, and stability

**Figure 9:**
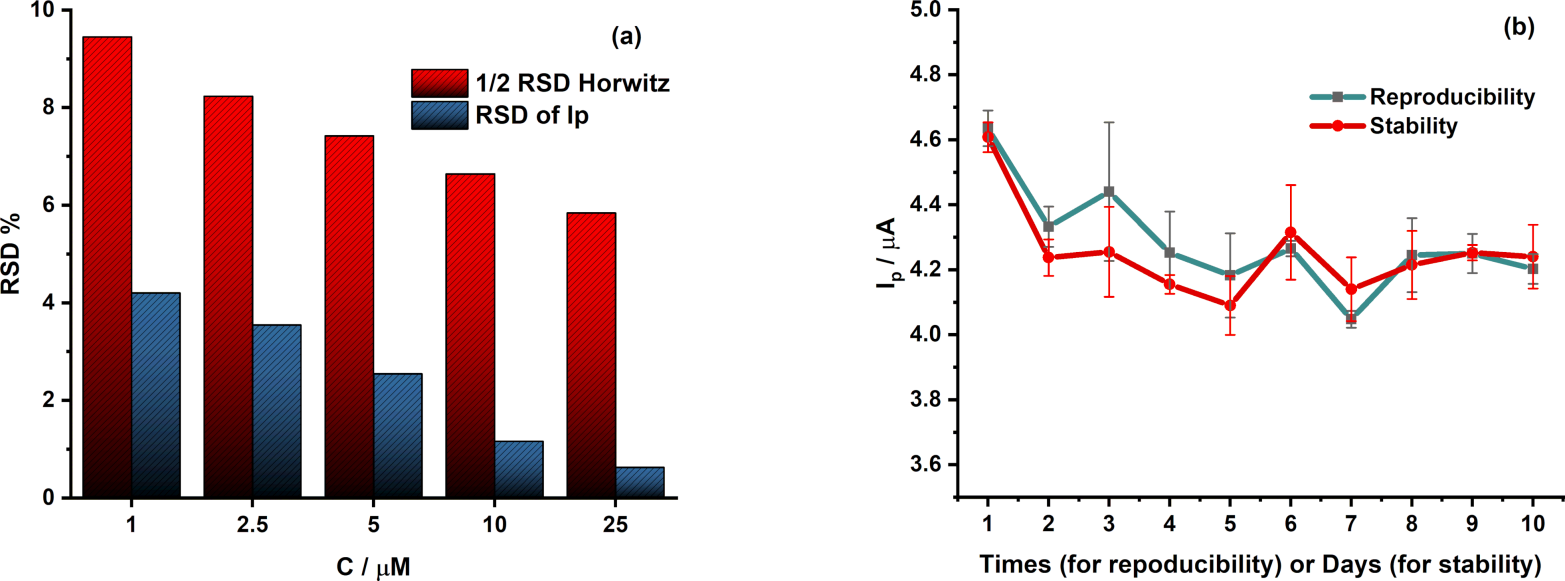
(a) Relative standard deviation of the anodic peak currents obtained from DPV measurements recorded over ten consecutive scans at the MIL-101/rGO(3.3)/GCE in 0.1 M B-R buffer (pH 4) containing CPR at different concentrations (1.0, 2.5, 5.0, 10.4, and 24.4 µM), in comparison with half of the Horwitz RSD (1/2 RSD_H_) and (b) reproducibility and long-term stability of the MIL-101/rGO(3.3)/GCE evaluated from the peak current response toward ciprofloxacin (5.0 µM) in 0.1 M Britton–Robinson buffer (pH 4), including ten repeated measurements using independently prepared electrodes and the current response recorded over ten days.

The measurement repeatability of the proposed sensor was evaluated at different CPR concentrations (1.0, 2.5, 5.0, 10.4, and 24.4 µM). Differential pulse voltammetry (DPV) responses were recorded over ten consecutive measurements, and the relative standard deviation (RSD) values were calculated and compared with half of the Horwitz RSD. The obtained RSD values were significantly lower than half of the Horwitz RSD, indicating excellent repeatability and analytical precision of the sensor (Figure 9a).

The reproducibility of the sensor was further assessed using independently prepared MIL-101/rGO-modified electrodes fabricated under identical conditions (Figure 9b). The peak current response was measured as *I*_CPR_ = 4.29 ± 0.16 µA, corresponding to an RSD of 3.70%. The low RSD value (<5%) confirms the good fabrication reproducibility of the proposed electrode.

The long-term stability of the sensor was evaluated using a single electrode stored in BR buffer at low temperature after use and tested over several days (Figure 9b). The peak current was recalled as *I*_CPR_ = 4.25 ± 0.14 µA with an RSD of 3.33%. After ten days of storage, approximately 92% of the initial current response was retained. The low RSD values together with the high current retention demonstrate the satisfactory long-term stability and reliable performance of the MIL-101/rGO-modified electrode.

#### Interference study

The selectivity of the proposed sensor toward ciprofloxacin (CPR) was evaluated in the presence of common inorganic ions and organic compounds, including CaCl_2_ (denoted as C1), NaCl (C2), K_2_SO_4_ (C3), Al(NO_3_)_3_ (C4), Mg_3_(C_6_H_5_O_7_)_2_ (C5), NH_4_Cl (C6), citric acid (C7), saccharine (C8), ᴅ-sorbitol (C9), ascorbic acid (C10), ᴅ-glucose (C11), and lactose (C12). An interfering species was considered to have a significant effect on the CPR peak current when the absolute relative deviation exceeded 5%.

**Figure 10:**
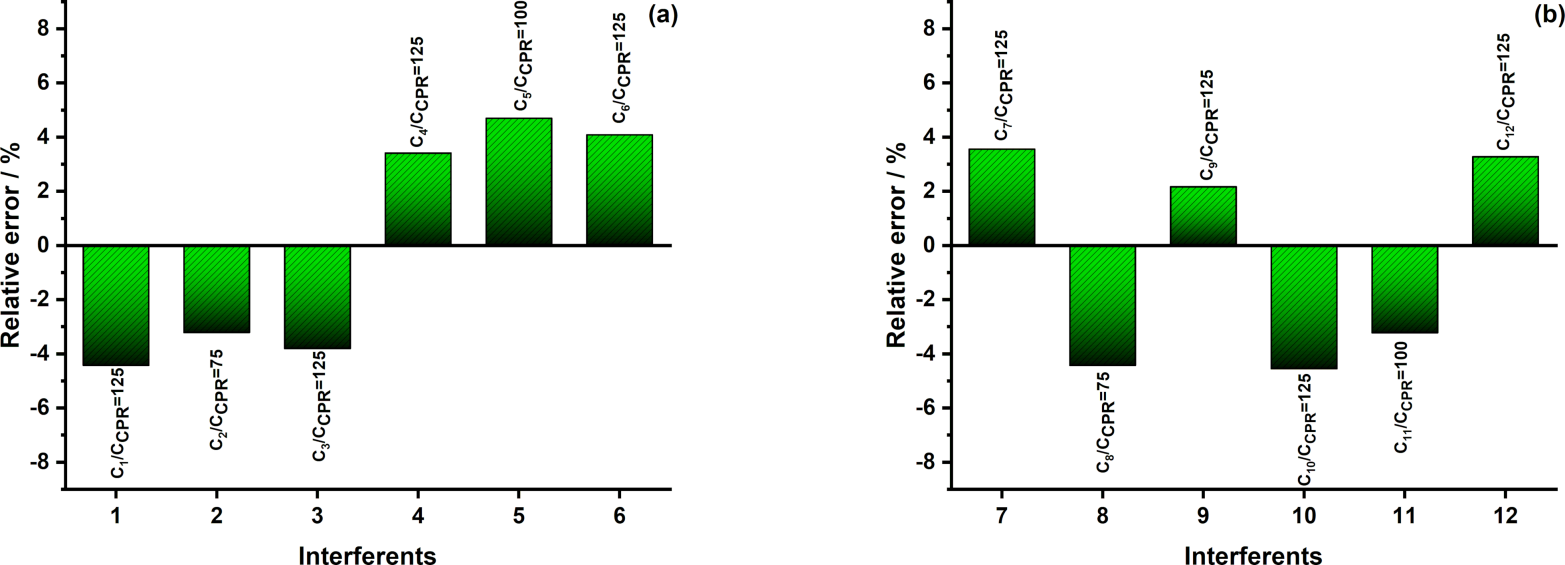
Relative error (RE%) caused by the interference of selected inorganic (a) and organic (b) species at different concentration ratios.

As shown in Figure 10, no significant interference was observed for most of the tested species, even when their concentrations were more than 100-fold higher than that of CPR. These results demonstrate that the proposed sensor possesses good selectivity and strong anti-interference capability, making it suitable for the determination of CPR in complex sample matrices.

#### Application to pharmaceutical samples

The proposed DPV method was used to determine CPR in various commercial pharmaceutical formulations using the standard addition method (Table 3). The recovery values obtained ranged from 95.91% to 104.67%, demonstrating good accuracy of the method. The slightly higher recovery for Tablets 2 (104.67%) may be due to excipients in the formulation. Some excipients can produce weak electrochemical responses or contribute to background currents near CPR oxidation potentials, which can slightly raise the measured peak current and cause a minor overestimation of the analyte concentration. However, these recoveries are within the acceptable range for pharmaceutical analysis (90–110%), confirming the reliability of the developed DPV method for analyzing CPR in real samples.

**Table 3:** Determination and recovery of CPR in commercial pharmaceutical formulations by DPV at MIL-101/rGO(3.3)/GCE.^a^

Notation	CPR content ± SD (µM)^b^	CPR content ± SD (mg)^c^	Spiked (µM)	Found ± SD (µM)^b^	Recovery (%)

Tablets 1 (Label 500 mg CPR/unit)	1.4376 ± 0.0182	479.3 ± 3.0	1.0	2.4089 ± 0.0299	97.13
Tablets 2 (Label 500 mg CPR/unit)	1.5656 ± 0.0281	512.1 ± 7.9	1.0	2.6123 ± 0.0158	104.67
Powder of oral suspension (Label 250 mg CPR/unit)	1.4851 ± 0.0172	245.6 ± 3.9	1.0	2.4442 ± 0.0257	95.91

^a^SD: standard deviation of three repeated measurements; ^b^CPR concentration calculated in electrochemical cell; ^c^CPR amount calculated in one unit of drug by DPV method.

## Conclusion

In this work, an electrochemical sensor based on a MIL-101/reduced graphene oxide (MIL-101/rGO) modified glassy carbon electrode was successfully developed for detecting ciprofloxacin. MIL-101/rGO, synthesized using a simple ultrasonic-assisted method, displayed a favorable microstructure and strong interfacial interaction between MIL-101 and rGO, leading to an improved electrochemical response to ciprofloxacin oxidation. Electrochemical investigations showed that the modified electrode exhibited enhanced electrochemical performance and increased catalytic activity toward CPR oxidation compared to the bare electrode. Under optimal experimental conditions, the proposed sensor demonstrated a broad linear response range with a low detection limit, along with good selectivity against common interfering substances. Additionally, the sensor exhibited excellent repeatability, strong reproducibility in fabrication, and reliable long-term stability. The practical application of the method was confirmed through successful detection of CPR in pharmaceutical products with acceptable recoveries. Overall, the MIL-101/rGO-modified electrode is a reliable and efficient electrochemical sensing platform for CPR detection and shows potential for routine pharmaceutical analysis and related electrochemical sensing research.

## Supporting Information

File 1 Additional figures.

## Data Availability

All data that supports the findings of this study is available in the published article and/or the supporting information of this article.
