# Supplementary material for: Electrochemical determination of ciprofloxacin using a MIL-101/reduced graphene oxide-modified electrode
Source: Beilstein J Nanotechnol. 2026 Apr 21;17:541–54. doi: 10.3762/bjnano.17.35 (PMC13159302; doi:10.3762/bjnano.17.35)
Supplement: File 1 — Additional figures. [file Beilstein_J_Nanotechnol-17-541-s001.pdf]

BEILSTEIN JOURNAL OF NANOTECHNOLOGY

Supporting Information

for

Electrochemical determination of ciprofloxacin using a MIL-101/reduced graphene oxide-modified electrode

Nguyen Quang Man, Nguyen Ngoc Nghia, Nguyen Vinh Phu, Vo Thi Khanh Ly, Le Lam Son, Pham Khac Lieu, Le Thi Hong Phong, Nguyen Dinh Luyen and Dinh Quang Khieu

Beilstein J. Nanotechnol. **2026,** *17,* 541–554. doi:10.3762/bjnano.17.35

Additional figures

License and Terms: This is a supporting information file under the terms of the Creative Commons Attribution License (https://creativecommons.org/licenses/by/4.0). Please note that the reuse, redistribution and reproduction in particular requires that the author(s) and source are credited and that individual graphics may be subject to special legal provisions.
The license is subject to the *Beilstein Journal of Nanotechnology* terms and conditions: (https://www.beilstein-journals.org/bjnano/terms)

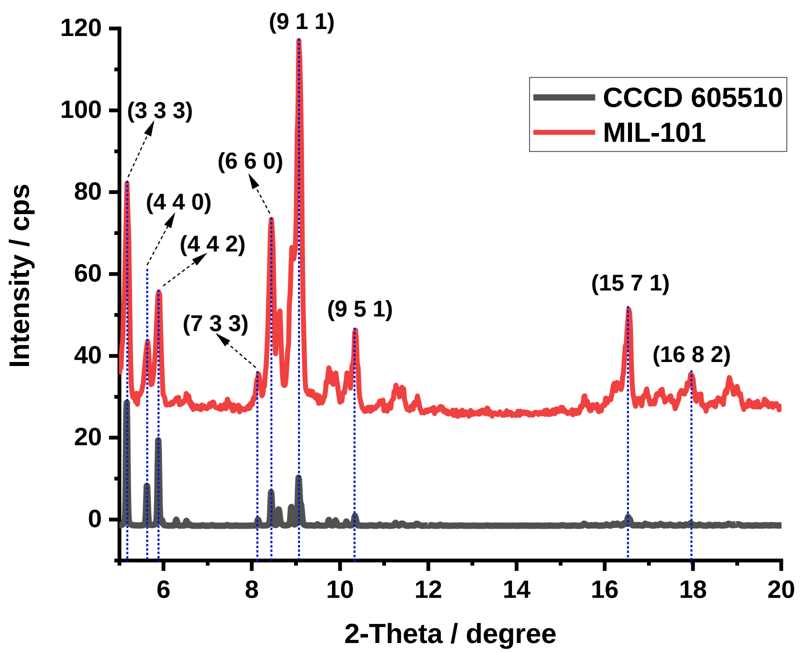

Figure S1: XRD pattern of the synthesized MIL-101

Peak indexing of MIL-101 based on the reference pattern (CCDC 605510 for MIL-101 (Cr)) shows that the diffraction peaks at ~5–10° correspond to the planes (333), (440), (442), (733), (660), (911), and (951), while the peaks at higher angles (~16–18°) are assigned to (15 7 1) and (16 8 2).

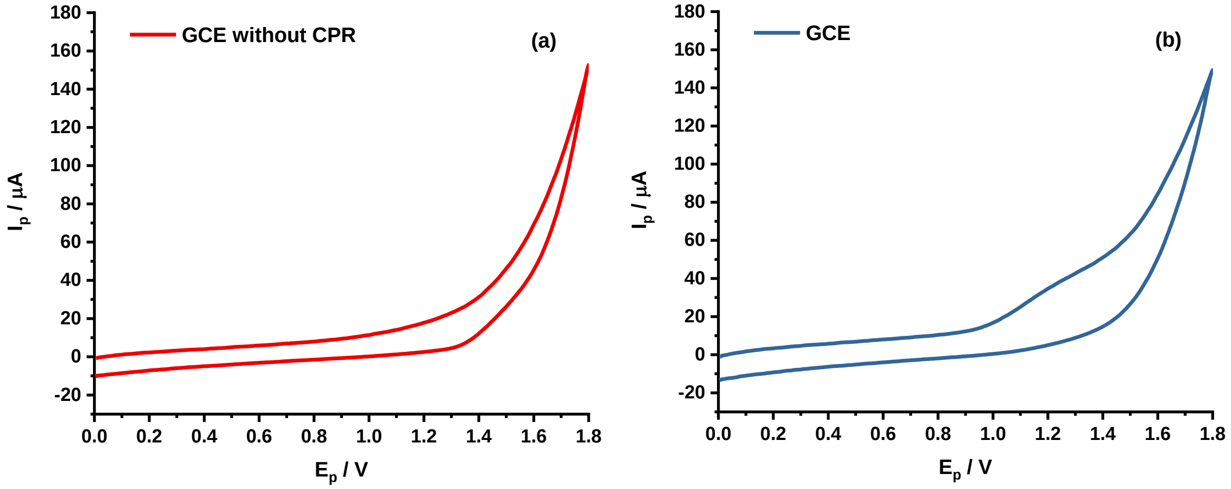

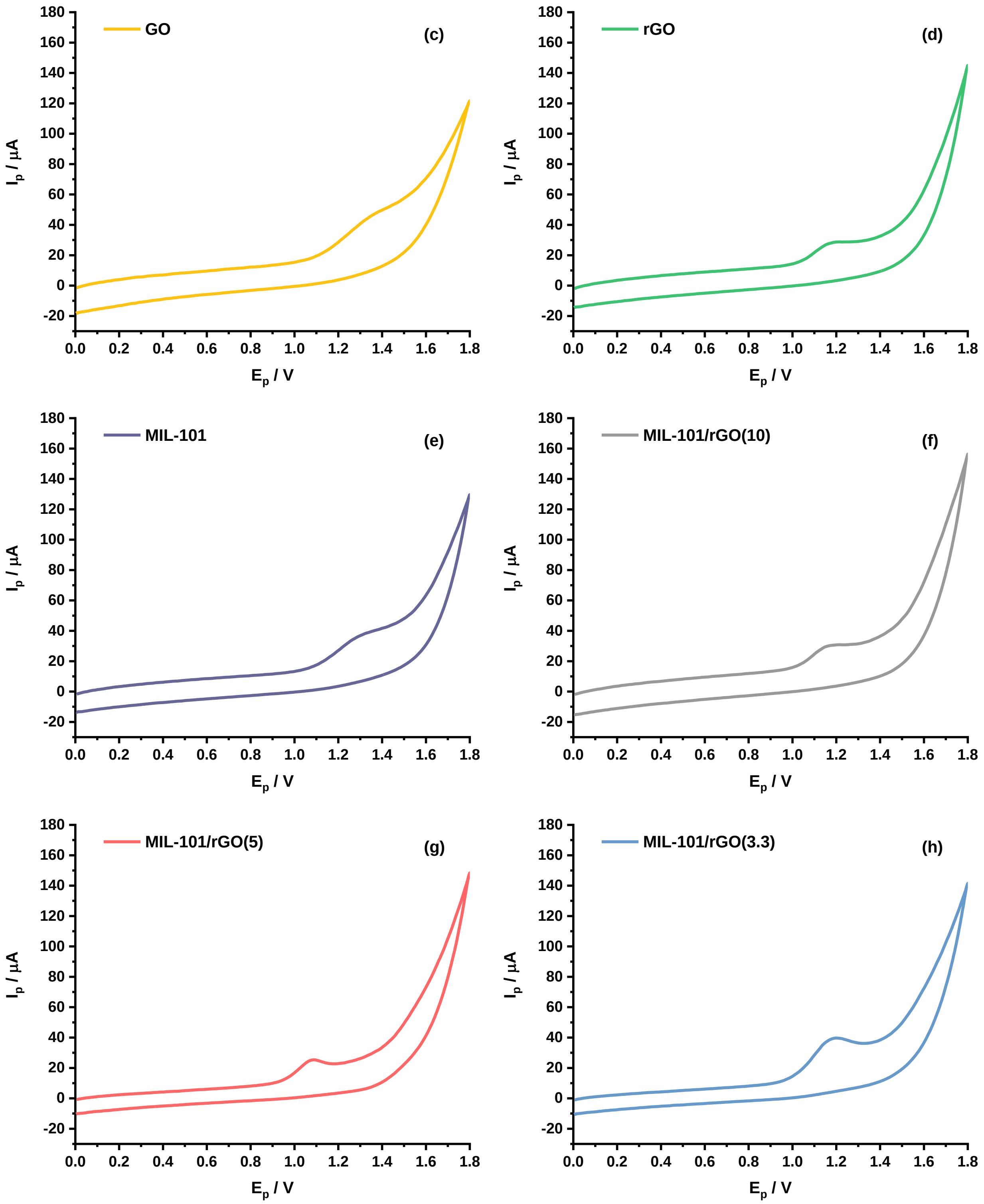
GO
(c)
rGO
(d)
MIL-101
(e)
MIL-101/rGO(10)
(f)
MIL-101/rGO(5)
(g)
MIL-101/rGO(3.3)
(h)
I_p / µA
E_p / V
180
160
140
120
100
80
60
40
20
0
-20
0.0
0.2
0.4
0.6
0.8
1.0
1.2
1.4
1.6
1.8

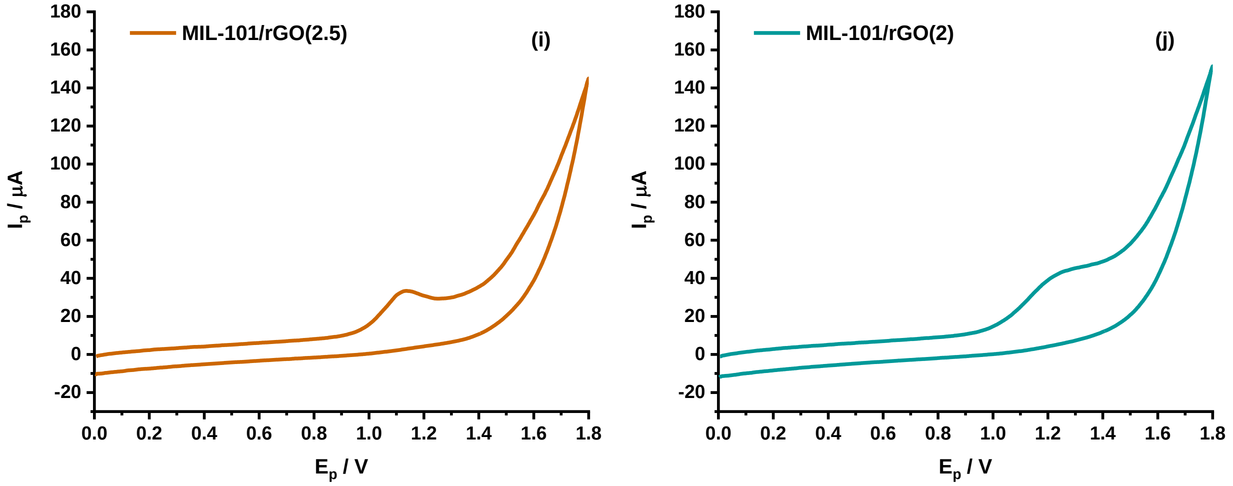

Figure S2: (a) Cyclic voltammograms (CVs) recorded at different electrodes in 0.1 M BRS buffer (pH 4) containing 15 µM CPR at a scan rate of 0.1 V/s.

The effect of the composition ratio of MIL-101 to rGO was also investigated. Among the tested materials, the composite with a MIL-101/rGO ratio of 3.3 exhibited the highest electrochemical response, indicating that this ratio provides the most favorable balance between the porous adsorption capability of MIL-101 and the high electrical conductivity of rGO. Therefore, the MIL-101/rGO (3.3) composite was selected as the optimal material for subsequent electrochemical measurements.

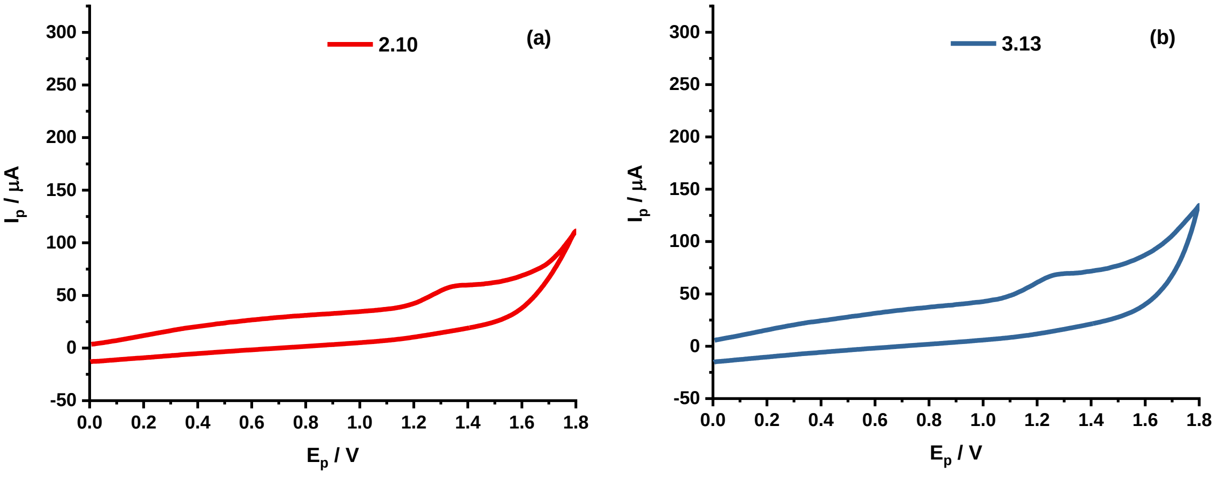

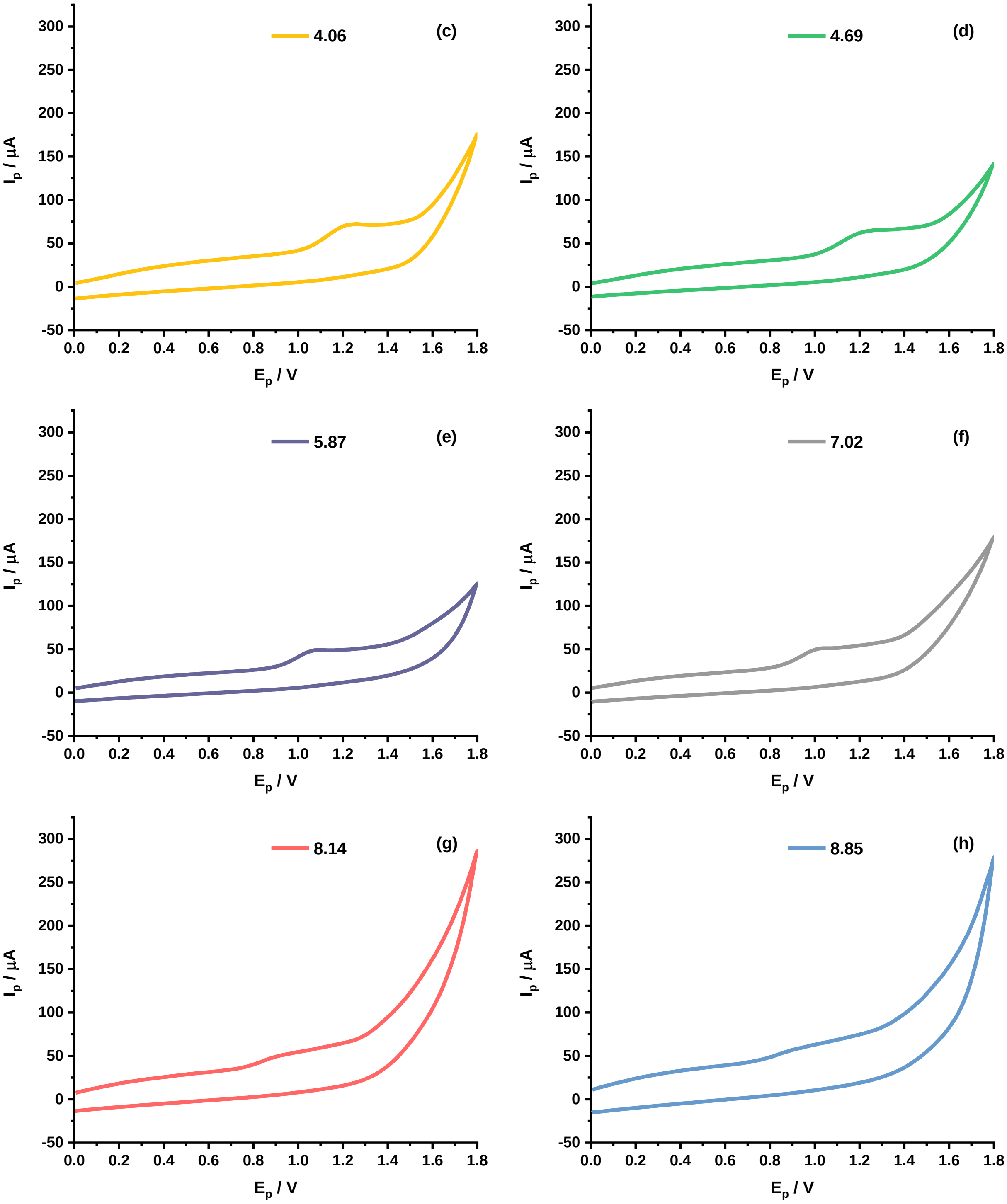

Figure S3: (a) CVs of MIL-101/rGO(3.3)/GCE recorded in 0.1 M BR buffer over the pH range of 2–9 containing 15 µM CPR at a scan rate of 0.1 V s^{-1}.

The effect of pH on the electrochemical response of CPR was investigated using CV at the MIL-101/rGO(3.3)/GCE in 0.1 M BR buffer with pH values ranging from 2 to 9 (Figure S3). The

peak current of CPR gradually increased with increasing pH and reached the maximum at pH 6, indicating the most favorable electrochemical oxidation conditions at this pH. When the pH was further increased above 6, the peak current slightly decreased, which may be attributed to changes in the protonation state of CPR and the electrode surface interactions. Therefore, pH 6 was selected as the optimal pH for subsequent electrochemical experiments.

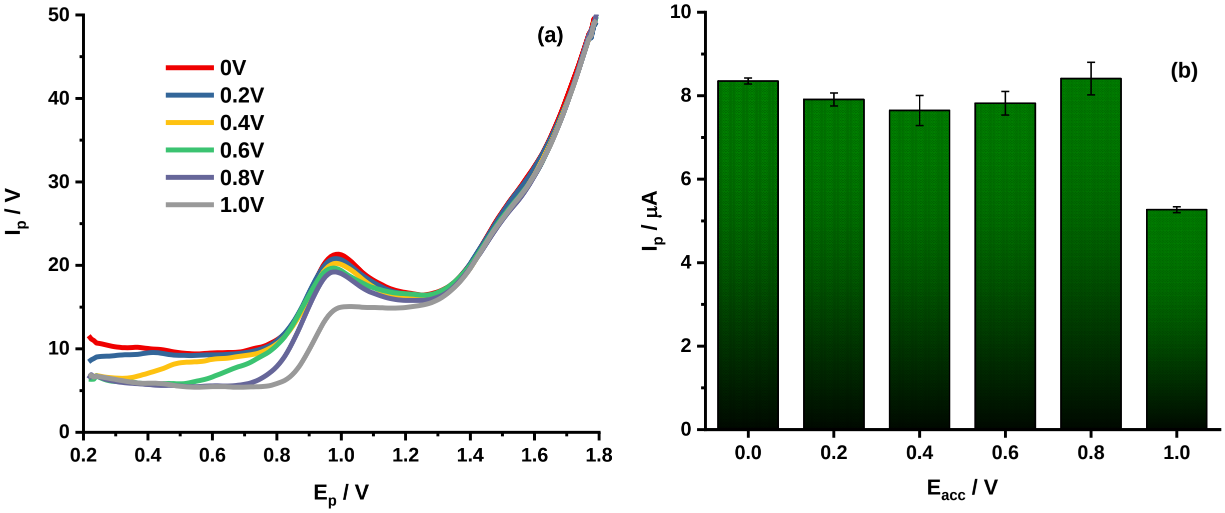

Figure S4: DPV curves of MIL-101/rGO(3.3)/GCE in BRS 0.1 M (pH = 4) with C_{CPR} = 15 µM in the accumulation potential range from 0 V to +1.0 V (accumulation time: 4 s; pulse amplitude: 0.08 V; voltage step: 0.008 V).

The influence of accumulation potential on the electrochemical response of CPR was investigated in the potential range from 0 to +1.0 V under the optimized experimental conditions (Figure S4). The results show that the peak current does not change significantly within the studied potential range, indicating that the accumulation potential has a negligible effect on the electrochemical response of CPR at the MIL-101/rGO(3.3)/GCE. Therefore, an accumulation potential of 0 V was selected for subsequent measurements due to its simplicity and stable response.

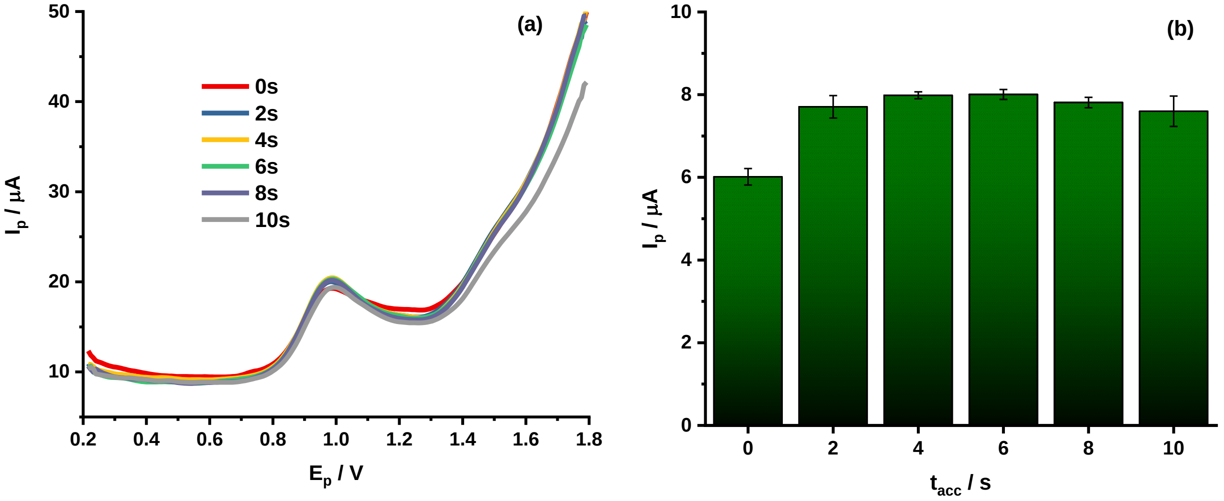

Figure S5: DPV curves of MIL-101/rGO(3.3)/GCE in BRS 0.1 M (pH=4) with C_{CPR} = 15 μM, the accumulation time from 0 to 10 s (accumulation potential: 0 V; pulse amplitude: 0.08 V; voltage step: 0.008 V).

Figure S5 shows the influence of accumulation time (0–10 s) on the DPV response of 15 μM CPR at the MIL-101/rGO(3.3)/GCE in 0.1 M BRS (pH 4). The peak current increases from 0 to 4 s and then remains nearly constant with further increase in accumulation time, indicating saturation of adsorption sites. Therefore, 4 s was selected as the optimal accumulation time.

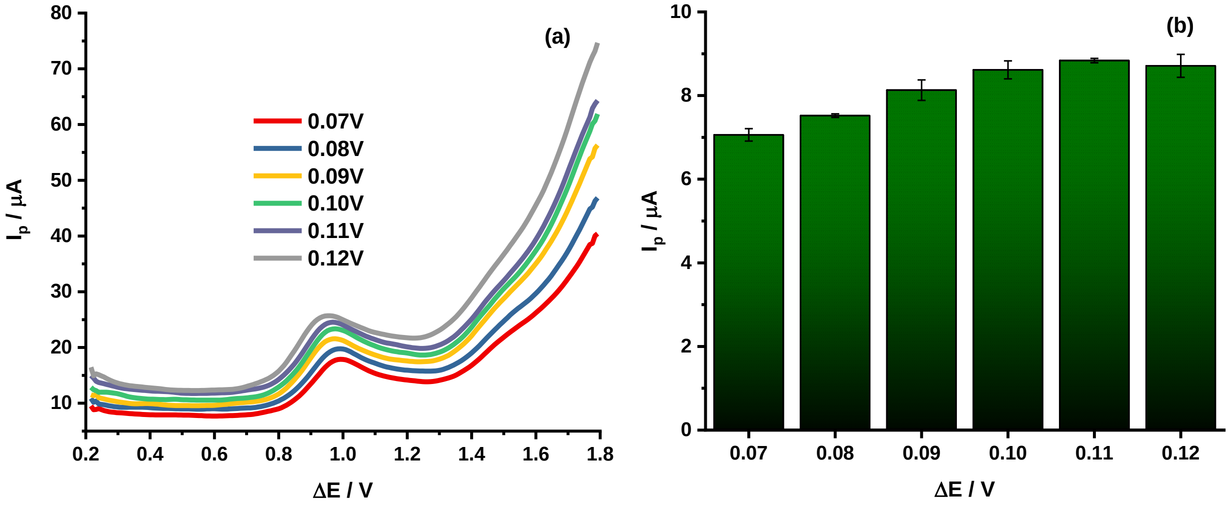

Figure S6: DPV curves of MIL-101/rGO(3.3)/GCE in BRS 0.1 M (pH=4) with C_{CPR} = 15 μM, pulse amplitude from 0.07 to 0.12 V (accumulation potential: 0 V; accumulation time: 4 s; voltage step: 0.008 V)

The effect of pulse amplitude on the DPV response of CPR at MIL-101/rGO(3.3)/GCE was studied in 0.1 M BRS (pH 4.0) containing 15 µM CCPR (Figure S6). The pulse amplitude was varied from 0.07 to 0.12 V. The peak current increased as the pulse amplitude increased; therefore, 0.11 V was chosen as the optimal value for future measurements.

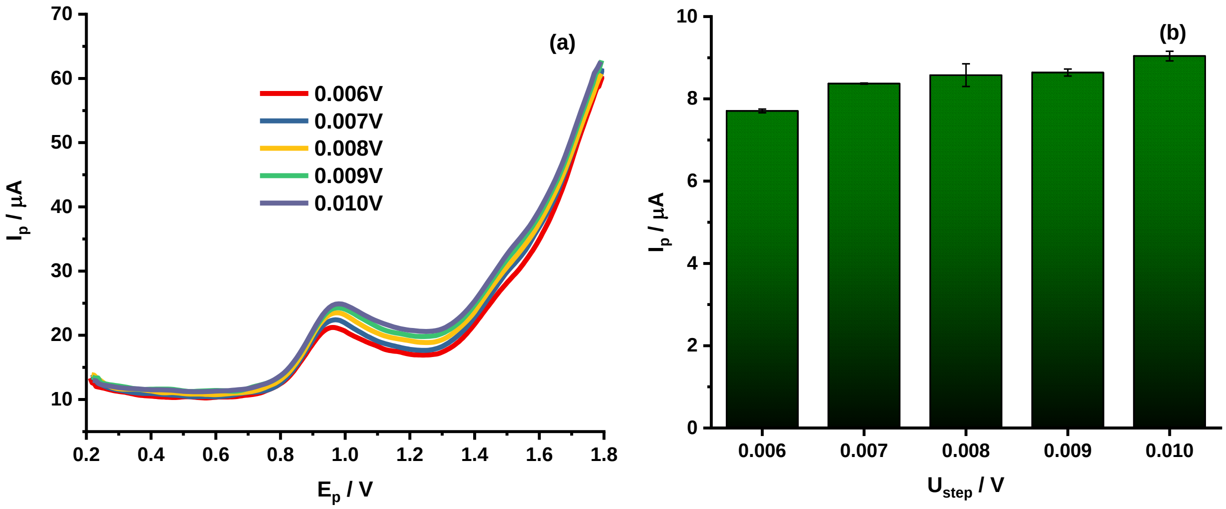

Figure S7: DPV curves at MIL-101/rGO(3.3)/GCE in BRS 0.1 M (pH=4) with C_{CPR} = 15 µM, accumulation potential: +0 V, accumulation time: 4 s, pulse amplitude: 0.11 V; voltage step from 0.006 to 0.010V.

The effect of the voltage step was examined from 0.006 to 0.010 V (Figure S7). The peak current increased slightly with increasing voltage step, while the peak potential showed negligible change. Considering the balance between signal intensity and peak resolution, 0.01 V was selected for further experiments.
